# CD40L induces multidrug resistance to apoptosis in breast carcinoma and lymphoma cells through caspase independent and dependent pathways

**DOI:** 10.1186/1471-2407-6-75

**Published:** 2006-03-18

**Authors:** Nathalie Voorzanger-Rousselot, Laurent Alberti, Jean-Yves Blay

**Affiliations:** 1 Equipe Cytokines et Cancer, Unité INSERM U590, Centre Léon Bérard, 28 rue Laënnec, 69373 LYON cedex 08, France

## Abstract

**Background:**

CD40L was found to reduce doxorubicin-induced apoptosis in non Hodgkin's lymphoma cell lines through caspase-3 dependent mechanism. Whether this represents a general mechanism for other tumor types is unknown.

**Methods:**

The resistance induced by CD40L against apoptosis induced by a panel of cytotoxic chemotherapeutic drugs in non Hodgkin's lymphoma and breast carcinoma cell lines was investigated.

**Results:**

Doxorubicin, cisplatyl, etoposide, vinblastin and paclitaxel increased apoptosis in a dose-dependent manner in breast carcinoma as well as in non Hodgkin's lymphoma cell lines. Co-culture with irradiated L cells expressing CD40L significantly reduced the percentage of apoptotic cells in breast carcinoma and non Hodgkin's lymphoma cell lines treated with these drugs. In breast carcinoma cell lines, these 5 drugs induced an inconsistent increase of caspase-3/7 activity, while in non Hodgkin's lymphoma cell lines all 5 drugs increased caspase-3/7 activity up to 28-fold above baseline. Co-culture with CD40L L cells reduced (-39% to -89%) the activation of caspase-3/7 induced by these agents in all 5 non Hodgkin's lymphoma cell lines, but in none of the 2 breast carcinoma cell lines. Co culture with CD40L L cells also blocked the apoptosis induced by exogenous ceramides in breast carcinoma and non Hodgkin's lymphoma cell lines through a caspase-3-like, 8-like and 9-like dependent pathways.

**Conclusion:**

These results indicate that CD40L expressed on adjacent non tumoral cells induces multidrug resistance to cytotoxic agents and ceramides in both breast carcinoma and non Hodgkin's lymphoma cell lines, albeit through a caspase independent and dependent pathway respectively.

## Background

Primary or secondary chemoresistance to cytotoxic chemotherapy is a frequent phenomenon in patients with solid or hematological malignant tumors and is a major cause of death of these patients. The most common cytotoxic agents used for the treatment of advanced cancers act by inducing the apoptosis of tumor cells through activation of the caspase cascade [1-4], although caspase independent pathways have been also reported [5-7]. The understanding of the mechanisms by which tumor cells become resistant to apoptosis is therefore an important issue to develop strategies to counteract tumor cell resistance to cytotoxic agents.

The acquisition by tumor cells of resistance to the apoptosis induced by cytotoxic drugs involves various biological mechanisms shared by tumors of different histological types [8-11,3]. These include the amplification of genes encoding for the enzymatic target of the cytotoxic agent, e.g. dihydrofolate reductase (DHFR) for methotrexate (MTX), or the up-regulation of transmembrane molecules capable to transport the drug outside the cell [9-11]. However, there is no consistent correlation between the expression of these proteins and the onset of drug resistance in vivo, suggesting the presence of additional biological mechanisms of drug resistance in vivo in cancer patients [11,12].

Among these, several observations indicate that integrins may protect tumor cells against the cytotoxic effects of anticancer agents after interaction with glycoproteins expressed in the extra cellular matrix or at the surface of adjacent non tumoral cells [13,14]. In a previous report, we showed that CD40L, a glycoprotein expressed normally on activated T lymphocytes, inhibits the cytotoxic and cytostatic effect of doxorubicin (DOX) by inhibiting caspase-3 activation in B lymphoma cell lines (NHL) [15,16]. Similar observations have also been made in chronic lymphocytic leukemia [17,18]. CD40L interacts with CD40, a transmembrane molecule of the TNF receptor family [19]. Physiologically, CD40 is expressed on normal B lymphocytes, interacts with CD40L expressed on activated T cells [19] and exerts a complex modulation of B cell apoptosis: CD40 promotes the survival of germinal center B cell, but also induces Fas expression thereby rendering the cells sensitive to FasL or agonists [19-21]. A similar situation is observed in neoplastic CD40 expressing B lymphoma cells, where CD40L has been reported to promote either cell survival or tumor regression [21-30].

CD40 is expressed not only on cells of the hematopoietic system but also on melanoma and on epithelial cells, in particular breast, lung, or ovarian carcinoma cell lines [31-37]. As for B lymphocytes, breast carcinoma cell survival is affected by CD40L in a complex manner: soluble CD40 ligand has been reported to inhibit the growth of breast carcinoma cell lines while membrane bound CD40L enhances Fas mediated apoptosis; conversely, stimulation of CD40+ breast carcinoma cells inhibited paclitaxel-induced apoptosis [32,35,38,39]. Therefore, CD40 signaling may either protect or enhance the apoptotic signal in tumor cells. The effect of CD40L on the apoptosis induced by cytotoxic agents in breast carcinoma was poorly studied [38] and no mechanistic studies have been reported.

In the present report, we investigated the capacity of CD40L to modulate apoptosis induced by a variety of cytotoxic agents with different modes of action in breast and NHL cell lines. The results indicate that CD40L induces a multidrug resistance to apoptosis in both breast cancer cell lines and NHL cell lines, through caspase independent and dependent pathways.

## Methods

### Cell lines and culture conditions

Lymphoma cell lines (DAUDI, RAJI, BJAB, BL36, BL70), renal carcinoma (CHA, MET, GUI, VER, TUM T, TUM G) cell lines [40], HTB81 prostatic carcinoma and the HCT116 colon carcinoma cell lines were grown at 2 × 10^5 ^to 10^6 ^cells/ml in RPMI 1640 (Life Technologies, Gibco BRL, Cergy Pontoise, France) for NHL cell lines and carcinoma, containing 10% fetal calf serum (FCS), 100 U/ml penicillin, 100 mg/ml streptomycin and 2 mM L-glutamin (Life Technologies, Gibco BRL, Cergy Pontoise, France). Breast carcinoma cell lines (T47 D, BT20, MCF-7) were grown at 10^5^–-2 × 10^5 ^cells/ml in DMEM (Life Technologies, Gibco BRL, Cergy Pontoise, France). The MCF-7 cell line did not express detectable levels of CD40 and was therefore used as negative control for CD40/CD40L effect. CDw32/FcγRII and CD40 ligand (CD40L) transfected Ltk (-) cell lines (CDw32 L cells and CD40L L cells) were kindly provided by Schering-Plough (Dardilly, France). The former being the negative control to the latter, FcγRII had no counterpart on NHL and BCC cell lines. 95% of the transfected L cells expressed CD40L as previously reported [15]. Cytotoxic agents (see under) were added at the initiation of the culture during 24 hours. After 24 hours, the medium was removed and replaced with the same culture medium without drugs for 24 additional hours of culture. Irradiated (75 Gy) transfected L cell lines were added 24 hours before the initiation of drugs exposure at a ratio of 1/10 L cells/tumor cell. Apoptosis, proliferation test and caspase activation were performed 72 hours after the initiation of tumor cell culture.

### Drugs and reagents

Doxorubicin (DOX) were purchased from Pharmacia (Paris, France), vinblastine (VIN) from Lilly France SA (Saint-Cloud, France), etoposide (ETO) from Pierre Fabre (Castre, France), Cisplatin (CDDP) from Rhône-Poulenc-Rorer Pharma (Antony, France), Paclitaxel (taxol, TAX) from Bristol-Myers-Squibb (Paris, France), D-erythro-sphingosine, N-acetyl-C2 ceramide (C2), D-erythro-sphingosine, N-hexanoyl-C6 ceramide (C6) and the control C2-dihydroceramide N-acetyldihydrosphingosine (C-) were purshased from Calbiochem (Meudon, France) and stock solutions were prepared in ethanol. Cell permeable inhibitors of caspase-9, 8 and 3-like activities (Z-LEHD-FMK: C9, Z-IETD-FMK: C8, Z-DEVD-FMK: C3) (R&D System GmbH, Abingdon, United Kingdom) were added at the initiation of cell culture, 24 hours before cytotoxic agents. Caspases inhibitors were used by different authors [41,42] between 50 and 200 μM depending on cell type and the observed phenomenon like apoptosis, cell cycle arrest and PARP cleavage. In our study we used inhibitors at 100 μM. The monoclonal anti CD44 antibody was purchased from Immunotech (Marseille, France).

### Determination of apoptosis

According to several publications the effects of drug may vary depending on cell type and drug concentration [43-48]. We used drugs at concentrations which induced apoptosis, i.e. for DOX: 0.5 μg/ml, ETO: 7,5 μg/ml, CDDP: 7,5 μg/ml, VIN: 50 nM, TAX: 50 nM, C2 and C6 ceramides: 75 μM. NHL cells were removed from 24-wells microtiter plates using gentle aspiration to avoid the removal of adherent CD40L L cells. A phenotypic analysis of the NHL fraction was performed for each experiments (CD20 and size) [15]. BCC lines were harvested after incubation with 1% trypsine, washed and resuspended 2 hours in medium. Indirect CD44 phycoerythrin labelled was then performed to distinguish breast carcinoma cell lines (CD44+) from irradiated CD40L L expressing L cells (CD44-). The quantification of apoptosis was previously performed using TUNEL assay (Boehringer Mannheim Corporation, Indianapolis, USA). Cell lines were fixed with 1% paraformaldehyde, permeabilised with 0.1% triton ×100 in 0.1% sodium citrate and washed extensively. Incubation with terminal deoxynucleotidyl transferase (TdT) and fluorescein-labeled d-UTP provided visualization of DNA strand breaks by flow cytometry on a FACScan instrument (Becton Dickinson, Pont de Claix, France). In each condition, 2000 cells were evaluated for their content in fluorescein labelled DNA strand breaks. The intensity of fluorescence was proportional to the number of fluorescein labelled DNA strand breaks. The threshold level of fluorescence intensity beyond which cells were considered to be in apoptosis was 10^1^.

### Proliferation assay

Thymidine incorporation in the different cell lines was tested in the presence of lower, cytostatic, concentrations of DOX (0.5 μg/mL), ETO (5 μg/ml), CDDP (5 μg/ml), VIN (30 nM), TAX (30 nM), C2 and C6 ceramides (30–50 μM). NHL or BCC cell lines (3.5 10^4 ^cells in 200 μL) were cultured in 96 wells flat-bottomed microtiter plates. After 24 hours of culture with drugs and 24 additional hours of culture without drugs, cells were pulsed with 1 mCi/well of [^3^H]TdR (25 Ci/mmol, Amersham, Les Ulis, France) for 18 hours. [^3^H]TdR incorporation was measured by tritium detector using standard liquid scintillation counting techniques on a β counter (Packard, Rungis, France). Of note, after irradiation, CD40L L cells did not interfere with the proliferation assay with no significant [^3^H]TdR incorporation.

### Assay for caspase-3/7 (Yama/CPP32/apopain) activity

Drugs were used at the same concentrations than in apoptosis experiments. After 72 h of culture, 10^6 ^cells were harvested, washed in phosphate-buffered saline (PBS) and then resuspended in the lysis buffer [5× buffer CSH, triton 0.01%, orthovanadate 1×, protease inhibitor 1×] at 4°C for 30 min and finally centrifuged at 4°C for 15 min at 13 000 g. Caspase-3/7 activity was measured using caspase-3/7 cellular activity assay kit plus (Biomol, TEBU, Le Perray-En-Yvelines, France). OD's measurements were performed at 0, 30, 60, 90, 120, 150, 180 min at 405 nm. Caspase-3/7 activity was calculated with the following formula:

pmol/min = Slope (OD/min) × conversion factor (μM/OD) × assay volume (μl) where conversion factor is: 50 μM/Average A405 nm (OD of p-nitroanaline).

### Western blotting

Total protein extraction was obtained by resuspended treated cells (in the same conditions of apoptosis induction), in lysis buffer CSH (Tris 50 mM pH 7.4, Nacl 2.5 M, EDTA 5 mM, NaF 50 mM, triton 0.1%, orthovanadate 10 μM) and protease inhibitors cocktail from SIGMA (SIGMA-Aldrich, Saint-Quentin Fallavier, France) (PMSF 10 μg/ml, leupeptine 0.2 μg/ml, aprotinine 0.2 μg/ml, TPCK 2 μg/ml).

Protein extracts (30–50 μg) were heated 5 min at 100°C in loading buffer (SDS 2%, β mercaptoethanol 100 mM, Tris pH6.8 50 mM, glycerol 10%, bromophenol blue 0.1%). Denatured samples were run on a 8% acrylamide gel. After transfer onto PVDF membranes (Millipore, Saint-Quentin en Yvelines, France), the membranes were blocked overnight with blocking buffer: I-Block 0.2% (Tropix, Courtaboeuf, France), PBS 1× and Tween-20 0.1%. Then, the membranes were incubated with the first antibody anti-Poly (ADP-Ribose)-Polymerase (Roche, Meylan, France) at 1/1000, washed and incubated with alkaline phosphatase-conjugated secondary antibody. The reaction was revealed with chemoluminescence detection method with CSPD substrate (Roche, Meyland, France).

### Statistics

Statistical analyses were performed using paired Student t test.

## Results

### CD40L induces multidrug resistance to apoptosis in carcinoma and lymphoma cell lines

**Figure 1:**
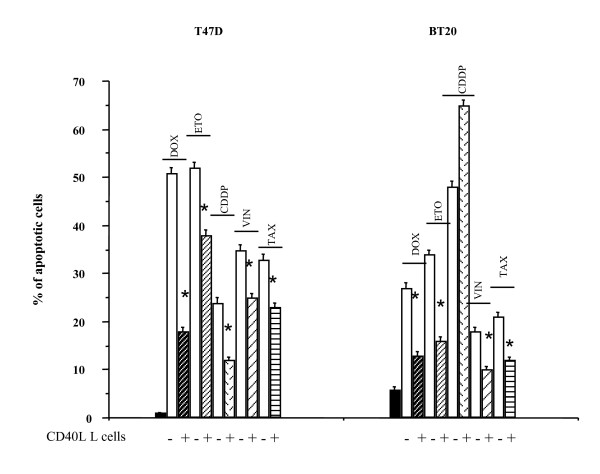
**Inhibition of drugs-induced apoptosis by CD40L L cells in carcinoma cell lines**. Breast carcinoma cell lines (T47D, BT20) were pre-incubated 24 hours alone or with irradiated (75 Gy) L cells expressing CD40L, then incubated with DOX (0.5 μg/ml), ETO (7.5 μg/ml), CDDP (7.5 μg/ml), VIN (50 nM) and TAX (50 nM) for 24 hours. The intensity of fluorescence, using TUNEL assay, was proportional to the number of fluorescein labeled DNA strand breaks. The threshold level of fluorescence intensity beyond which cells were considered to be in apoptosis was 10^1^. *Apoptosis was significantly decreased (Student t test, p < 0.05) upon co-culture with irradiated CD40L L cells as compared to no L cells This experiment is representative of 7 different experiments.

CD40 was found detectable in 2 of the 3 breast carcinoma cell lines (BCC), in all 6 renal cell carcinoma lines (RCC), in the HTB81 prostatic carcinoma and the HCT116 colon carcinoma cell lines as well as in 5 of 5 NHL cell lines tested (not shown). We investigated whether the apoptosis induced by a panel of cytotoxic agents with various modes of action (doxorubicin (Dox), paclitaxel (TAX), vinblastin (VIN), etoposide (ETO), cisplatin (CDDP) was affected by co-culture with CD40L expressing L cells (CD40L L cells). All 5 drugs tested induced the apoptosis of the 2 breast carcinoma cell lines (Figure 1). DOX increased apoptosis of the RCC (86% to 94%), prostatic (90%) and colon carcinoma cell lines (70%) tested. In the presence of CD40L L cells, the percentage of cells undergoing apoptosis after exposure to the 5 cytotoxic agents was significantly reduced (-23% to -62%) in the 2 BCC lines as compared to a co-culture with L cells expressing CDw32 (CDw32 L cells) (not shown) or without CD40L L cells (Figure 1). The only exception was observed with CDDP for the BT20 cell line. Of note, drugs induced apoptosis in CD40 (-) BCC MCF-7 but no protection could be observed in the presence of CD40L L cells (not shown). Co-culture with CD40L L cells also reduced the percentage of cells undergoing apoptosis in 4 of the 6 RCC cell lines exposed to DOX (-44% to -70%) and, marginally, in the HCT116 colon carcinoma cell line (-15%) and prostatic carcinoma cell line HTB-81 (-30%). Similarly, CD40L L cells significantly reduced the percentage of NHL cells undergoing apoptosis after treatment with DOX, CDDP and VIN in all 5 NHL cell lines, and after treatment with ETO, VIN and TAX in 3 to 4 of the cell lines tested (Table 1).

**Figure 2:**
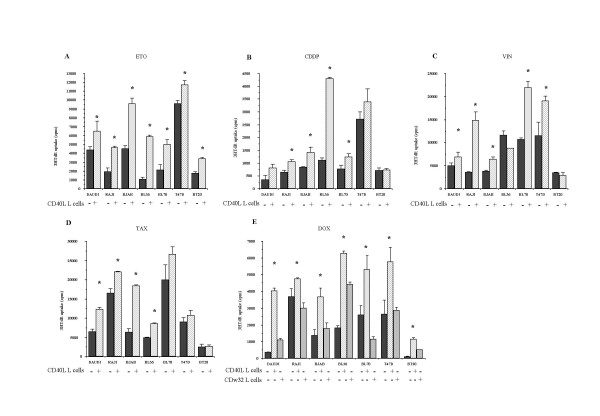
**CD40L L cells inhibit the cytostatic effects of anticancer drugs**. Cells were incubated with (A) ETO (7,5 μg/ml), (B) CDDP (7,5 μg/ml), (C) VIN (50 nM), (D) TAX (50 nM), (E) DOX (0.5 μg/mL), in the presence or absence of irradiated CD40L L cells or irradiated CDw32 L cells (in panel E), then washed and cultured without drugs but otherwise in the same conditions during 24 additional hours before measurement of [^3^H] TdR incorporation. *: [^3^H] TdR incorporation was significantly increased (Student t test, p < 0.05) upon co-culture with irradiated CD40L L cells as compared to no L cells, or L cells expressing CDw32 (E). These results are the mean and SE of triplicate and these results are representative of 4 different experiments.

In NHL cell lines, the cytostatic effect of all 5 drugs was partially reversed upon co-culture with CD40L L cells at 72 h (Figure 2) but also after 96 h, 168 h and 240 h of culture (not shown). In contrast, co-culture with CD40L L cells, but not with CDw32 L cells, also partially reversed the cytostatic effect of DOX and ETO in the 2 breast carcinoma cell lines at 72 h, and also after 240 h of culture (not shown), but did not affect the proliferation of breast carcinoma cell lines treated with CDDP, VIN and TAX (Figure 2). Since the drugs tested induced only a limited increase of the apoptosis in HTB-81 and RCC cell lines, and since the protective effect of CD40L on these two cell types were marginal, subsequent experiments were performed on breast carcinoma and lymphoma cell lines only.

### CD40L modulates the cytotoxic and cytostatic effects of ceramides on carcinoma and NHL cell lines

Ceramide are important mediators of the apoptosis induced by cytotoxic agents [49-52]. Incubation with ceramides C2 (N-acetyl-C2 ceramide) and C6 (N-hexanoyl-C6 ceramide) during 24 hours increased the apoptosis of the 2 breast carcinoma and the 5 NHL cell lines tested, while the negative control C2 dihydroceramide was inactive (Table 2). Co-culture with irradiated CD40L L cells reduced the percentage of apoptotic cells induced by ceramide exposure (75 μM) in all breast carcinoma (with a limited effect on T47D exposed to C6) and NHL cell lines as compared to cells cultured without CD40L L cells (Table 2). At lower concentrations (50 μM), ceramides blocked the proliferation of these cell lines: the anti-proliferative effect of C2 and C6 ceramides on the breast carcinoma and NHL cell lines tested was reversed by co-culture with CD40L L cells, except for C2 in BT20 breast carcinoma cell line and Daudi and BL70 NHL cell lines (Table 3).

### Modulation of caspase-3/7 activation by CD40L

DOX, ETO, VIN and TAX (but not CDDP) induced a modest increase (2 to 4-fold) of caspase-3/7 activity in the 2 breast carcinoma cell lines, while these 5 drugs (except CDDP in 4 NHL) almost consistently increased (up to 28-fold) caspase-3/7 activity in the 5 NHL cell lines tested (Table 4). This increase of caspase-3 activity was completely inhibited when cell lysates were pre-incubated with the inhibitor of caspase-3-like activity DEVD-FMK (not shown). In all 5 NHL cell lines, co-culture with CD40L L cells, but not with CDw32 L cells, inhibited (-39% to -89%) caspase-3/7 activity induced by DOX, ETO, VIN, TAX (Table 4). CD40L L cells significantly inhibited CDDP-induced caspase-3/7 activity in the BJAB cell line only (Table 4). In contrast, in breast carcinoma cell lines, co-culture with CD40L L cells did not significantly reduce caspase-3/7 activity, and actually consistently increased 2 to 4-fold caspase-3/7 activity as compared to cells treated with the same agents alone (Table 4).

### Modulation of PARP cleavage

**Figure 3:**
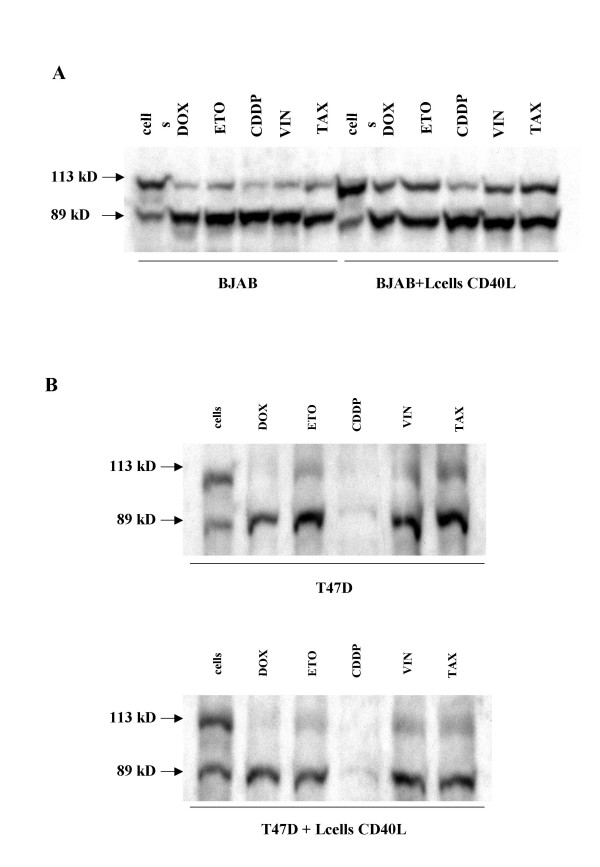
**Modulation of PARP cleavage by drugs and CD40L expressing L cells in NHL and BCC carcinoma cell lines**. BJAB cells (Panel A) and T47 D (Panel B) were incubated alone or with irradiated (75 Gy) L cells expressing CD40L then incubated with DOX (0.5 μg/ml), ETO (7.5 μg/ml), CDDP (7.5 μg/ml), VIN (50 nM) and TAX (50 nM) for 24 hours and finally washed and cultured without drugs with or without irradiated L cells expressing CD40L during 24 additional hours. Western blot were performed on 50 μg of total cell protein with PARP antibody. Data are representative of 3 experiments.

The modulation of caspase-3 activity was also investigated by the detection in Western Blot of caspase-3 products cleavage of PARP. During apoptosis, activated caspase-3 cleaves PARP (Poly-(ADP-Ribose)-Polymerase) (113 kD) into 89 kD and 24 kD fragments. In all 5 NHL and 2 BCC cell lines, drugs induced the appearance or an increase of the 89 kD fragment (Figure 3A, BJAB and 3B T47D), while the 113 kD form was dramatically reduced. Upon exposure of BJAB cell line to CD40L L cells, the full length form (113 kD) remained detectable, at level close to that of untreated BJAB cells (Figure 3A), although the 89 kD form remained present in cell lysates; similar observations were made in the other 4 NHL cell lines (not shown) for 4 of the 5 cytotoxic agents tested (not CDDP). For the two breast carcinoma cell lines T47D (Figure 3B), and BT20 (not shown) however, exposure to CD40L L cells did not significantly affect the balance of the 89 kD and 113 kD forms of PARP protein,; this observation is consistent with the absence of modulation of caspase-3 activity in the 2 breast carcinoma cell lines.

### Modulation of the apoptosis of the tumor cell lines by inhibitors of caspases-like activities

**Figure 4:**
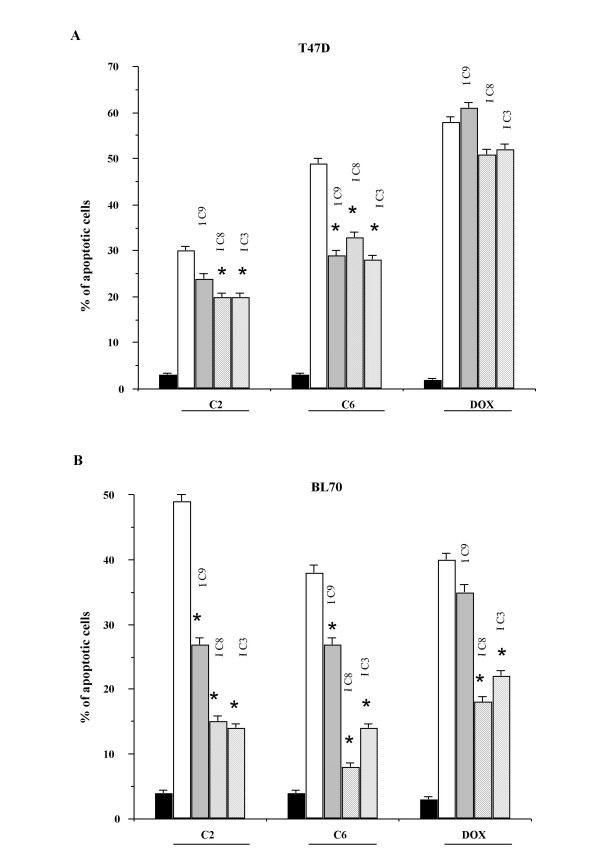
**Modulation of drug induced apoptosis by inhibitors of caspase like activities**. T47 D (Panel A) and BL70 cells (Panel B) were incubated alone; or 24 hours in the presence of C2 or C6 ceramides (75 μM) or DOX (1 μg/mL); or pre-incubated 24 hours with cell permeable inhibitors of caspase-9-like activity (z-LEHD) or caspase-8-like activity (z-IETD) or caspase-3-like activity (z-DEVD) and then 24 hours with drugs (C2 or C6 or DOX). Cells were finally tested for apoptosis using the TUNEL assay as indicated in Materials and Methods. *Apoptosis was significantly decreased (Student t test, p < 0.05) upon co-culture with caspases inhibitors as compared to no caspases inhibitors.

In order to determine whether caspase-3, and upstream caspases 8 and 9 played a role in drug-induced apoptosis, caspase-3, 8 and 9-like activities were blocked in drug-treated breast carcinoma and NHL cell lines using cell permeable tetrapeptide inhibitors. None of these 3 inhibitors blocked apoptosis induced by DOX in the T47D breast carcinoma cell lines (Figure 4A), nor in BT20 (not shown). Conversely, inhibitors of Caspase-3 and 8-like activities, but not of caspase-9-like activity, significantly reduced the percentage of apoptotic cells in BL70 treated with DOX (Figure 4B), as well as in the 4 other NHL cell lines (not shown). The role of endogenous ceramide in apoptosis remains unclear: these mediators may actually act upstream or downstream of caspases in different tumor models [53,54]. In the present study, inhibitors of caspase-3, 8 and 9-like activities protected both NHL and breast carcinoma cell lines from ceramide induced apoptosis (Figure 4), although the magnitude of protection observed in breast carcinoma cell line was limited in particular for C2 (Figure 4). Taken together, these results indicate that induction or protection from apoptosis by cytotoxic agents, ceramide and CD40L occur without consistent modulation of caspase-3, 8 and 9 activity in breast carcinoma cell lines.

## Discussion

CD40L is a member of the TNF family of ligand which is normally expressed by T lymphocytes and interacts with CD40 expressed on B cell and antigen presenting cells [19]. CD40L exerts complex anti- or pro-apoptotic effects in normal and transformed B lymphocytes, enhancing Fas mediated apoptosis [20,21] but protecting against apoptosis induced by cytotoxic agents [15-17]. CD40 expression is not limited to cells of the hematopoietic system, and has been found detectable on a variety of human carcinomas, including bladder, breast, ovarian, lung as well as in melanoma cell lines [31-37]. The purpose of this study was to investigate the effect of CD40L on the apoptosis of CD40-expressing carcinomas and lymphoma cell lines induced by a variety of cytotoxic agents.

In NHL cell lines, CD40 ligand expressed on adjacent non tumoral cells was found capable 1) to inhibit the apoptosis induced by five different commonly used cytotoxic agents (DOX, ETO, CDDP, VIN, TAX), 2) to inhibit the activation of caspase-3/7 induced by DOX, ETO, VIN and TAX, 3) to inhibit drugs (DOX, ETO, VIN TAX) induced PARP cleavage by apoptosis protease like YAMA/CPP32/Apopain/Caspase-3 and 4) to partially reverse the antiproliferative effect of the five cytotoxic agents in the 5 NHL cell lines tested. The inhibition of caspase-3-like and 8-like activities, but not caspase-9-like activity, by permeable tetrapeptide inhibitors also blocked the apoptosis induced by doxorubicin in NHL cell lines, suggesting that the downregulation of caspase-3-like is an essential molecular mechanism of the protective effect of CD40L.

However, the modulation of caspase-3 is not the sole mechanism of the protective effect of CD40L in NHL cell lines. Raji was found to be resistant to reversion of apoptosis induction by TAX and ETO despite of modulation of caspase-3-like activation and PARP cleavage. In addition, resistance by CD40L to CDDP induced apoptosis was not associated with caspase-3/7 and PARP cleavage modulation in 4 of the 5 NHL cell lines tested. Apoptosis resistance mechanisms acting downstream of caspase-3 activation have been described in particular in the Raji cell line [55,56] as well as in ovarian carcinoma cell lines treated with CDDP [57]. Therefore, protection from drug-induced apoptosis by CD40L in NHL cells occurs through a caspase dependent pathway for anthracyclins, etoposide, paclitaxel and vinblastin, but through a caspase independent pathway for CDDP and for the resistant Raji cell line, presumably at a convergent point of apoptosis induction for all cytotoxic agents downstream of caspase-3.

In contrast, in CD40 expressing breast carcinoma cell lines, the protective effect of CD40L was found to be caspase-independent. Co-culture with CD40L L cells was found capable to protect breast (as well as renal, prostatic and colon) carcinoma cell lines against DOX induced apoptosis. Exposure of the 2 breast carcinoma cell lines tested here to doxorubicin, CDDP, Paclitaxel, Vinblastin or etoposide did not or only weakly increased caspase-3/7 activity while caspase-3/7 activity increased up to 28-fold in NHL cell lines tested after exposure to the same drugs. Finally, CD40L did not inhibit caspase-3/7 activation and PARP cleavage induced by any of the 5 cytotoxic agents in the 2 breast carcinoma cell lines tested. Actually, exposure to CD40L L cell was found to increase caspase-3/7 activity in drug-treated breast cancer cells, in marked contrast with what was observed for NHL cell lines. Consistent with these observations, cell permeable tetrapeptide inhibitors of caspase-3, 8 and 9-like activities failed to inhibit the apoptosis of breast carcinoma cell lines, in contrast to what is observed in NHL cell lines. These results indicate 1) that caspase-3 activation is not the single pathway required for the induction of apoptosis of breast cancer cell lines by the anti-cancer drugs tested considering the PARP cleavage but the weak caspase-3/7 activity modulation by drugs and 2) that the anti-apoptotic effect of CD40L on these cell lines do not involve a modulation of caspase-3/7 activity as it was shown by the absence of inhibition of caspase-3 activity and PARP cleavage.

Different mechanisms for CD40L protection against drug-induced apoptosis and drug anti proliferative effect could be suggested. Some studies indicated that the antiapoptotic function of CD40 is mediated by up-regulated expression of bcl-xL gene, an antiapoptotic member of the bcl-2 family of proteins and that up-regulation of Bcl-xL could be a key event in CD40-mediated survival in both normal tonsillar B cells and the immature B-cell lymphoma WEHI-231 cells [58-60]. More recently, Lee et al provided a crucial link in CD40-mediated antiapoptosis by linking the activation of the NFKB-signalling pathway to the up-regulation of Bcl-2 family members [61]. The cytoplasmic domains of TNF receptor family like CD40 do not encode any enzymatic activity but some proteins, identified by the generic name TRAF, physically interact with them, providing some clues to the mechanisms of signal transduction [62]. The CD40 cytoplasmic domain has been found to interact with TRAF2, TRAF3, TRAF5 and TRAF6 but TRAF2 is the best characterized of the TRAF proteins in term of signaling function. TRAF2 activates NFκB by means of a synergistic interaction with a novel protein called TANK [63]. It has been suggested that TRAF2 would have to be released from CD40 in order for it to interact with TANK and activate NFκ B. Study of functional consequences, like NFkB activation, of preventing CD40-TRAF2 dissociation with the blocking antibody anti CD40L would provided information on mechanisms induced by CD40-TRAF2 interaction. Finally, the protective role of CD40L against drug-induced apoptosis may pass by the modulation of the cell cycle and the role of p53 family. Previous studies showed that CD40 can repress drug-induced apoptosis by, among others, B lymphoma cell cycle progression [64]. Teoh et al showed, depending on multiple myeloma cells p53 status, that CD40 induced increase G1/S transition and cell proliferation or growth arrest with sub G1 phase cells and apoptosis [65]. So many pathways remain to be explore in order to determine CD40/CD40L cellular mechanism.

Taken together, these results indicate that the protective effect of CD40L against cytotoxic agents involves caspase-dependent and independent pathways in NHL, and caspase independent pathways in breast carcinoma cells lines. Of note, similar observations were obtained in the colon carcinoma HCT116 cell line treated with doxorubicin, with no significant modulation of caspase-3/7 activity nor reversal of the cytostatic effect of doxorubicin (not shown). These results strongly suggest that the anti-apoptotic effect of CD40L involves different molecular mechanisms in the lymphoma and carcinoma cell lines tested in this study.

It has been reported that CD40L induces direct cytostatic effects on breast carcinoma cell lines [35,39]. The lack of significant impact of CD40L on tritiated thymidine incorporation in the 2 breast carcinoma cell lines tested could hence have resulted from a combined "direct" inhibitory effect of CD40L on BCC proliferation and a reversal of drug-induced cytostatic effect by CD40L. This hypothesis is however unlikely since no direct anti-proliferative effect of CD40L expressing L cells was observed in the two breast carcinoma cell lines tested (not shown). The discrepancy between the present and previously published results [35] regarding the effect of CD40L on breast carcinoma cell proliferation could result either from a variability of response to CD40L in different carcinoma cell lines or alternatively to a different biological activity of membrane bound vs soluble CD40L. Indeed, soluble and membrane bound CD40L, as well as CD40 agonists, have been reported to exert opposite biological activities on lymphoma cell line proliferation and survival in previous studies [15-17,23,27,28]. Tong et al reported apoptosis induction by CD40L in BCC in the absence of cytotoxic drugs, but moreover they used a recombinant molecule and not membrane bound CD40L [39]. On the other hand, in agreement with our current finding Stumm et al have shown that CD40 stimulation in Breast carcinoma inhibited drug-induced apoptosis [38].

Ceramides have been reported to act as second messengers for the apoptosis induced by DNA damaging agents in some tumor cell lines [49-52]. A rapid intracellular ceramide increase has been observed after exposure to γ radiation or exposure to DNA damaging agents, resulting either from the activation of a sphingomyelinase or ceramide synthase, or from caspase-8 activation in different tumor models [51]. To determine whether the protective signaI delivered by CD40L acts upstream or downstream of ceramide production, the protective effect of CD40L on the apoptosis induced by ceramides in BCC and NHL cell lines were compared. While cell permeable C2 and C6 ceramides induced apoptosis and blocked thymidine incorporation in the 5 NHL and 2 breast carcinoma cell lines tested, co-culture in the presence of CD40L L cells blocked the apoptotic signal induced by C2 and C6 ceramides in NHL and breast carcinoma cell lines. However, the molecular mechanisms involved in the induction of apoptosis by ceramides and cytotoxic agents were found to be different in breast carcinoma. While inhibitors of caspase-3 and 8-like activities partially prevented the apoptosis induced both by ceramide or doxorubicin in NHL cell lines, these inhibitors were active only for ceramide-induced apoptosis (and not for drug-induced apoptosis) in breast carcinoma cell lines. Since ceramide production in response to cytotoxic agents was not tested in the present study, it is not possible to establish whether CD40L affects ceramide increase in response to cytotoxic agents. However, ceramide and doxorubicin-induced apoptosis were modulated by different inhibitors in breast carcinoma suggesting that ceramide is at least not an exclusive mediator of doxorubicin induced apoptosis in this breast carcinoma cell line.

CD40L therefore may block the apoptosis of breast carcinoma cell lines independently of the modulation of caspase activities in these different models. Further studies on bcl-2 family protein like Bclxl/Bax, but also cell cycle, p53 and TRAF-2 would provide more information on mechanism of CD40L induced drug resistance.

## Conclusion

This study show that CD40L expressed on adjacent non tumoral cells induce a multidrug resistance to apoptosis in breast carcinoma and NHL cell lines, through both caspase dependent and independent pathways in NHL cell lines, and through a caspase independent pathway in breast carcinoma cell lines.

## Abbreviations

NHL = non Hodgkin's lymphoma; BCC = breast carcinoma cell lines; DOX = doxorubicin; ETO = etoposide; CDDP = Cisplatin; VIN = vinblastin; TAX = paclitaxel; C2 = D-erythro-sphingosine, N-acetyl-C2 ceramide; C6 = D-erythro-sphingosine, N-hexanoyl-C6 ceramide; C- = C2-dihydroceramide N-acetyldihydrosphingosine; PARP = Poly (ADP-Ribose)-Polymerase.

## Competing interests

The author(s) declare that they have no competing interests.

## Authors' contributions

NVR carried on quantification of apoptosis by flow cytometry, proliferation assays, caspase-3/7 activity determination. LA helped for Western blotting. JYB participated in the design and coordination of the study and helped to draft manuscript. All authors read and approved the manuscript.

## Pre-publication history

The pre-publication history for this paper can be accessed here:



## Figures and Tables

**Table 1:** Multidrug resistance induced by CD40L in lymphoma cell lines

**Culture conditions**		**% of apoptotic cell Mean (SE)**				
		**LNH**				
**Drug**	**L cells CD40L**	**DAUDI**	**Raji**	**BJAB**	**BL36**	**BL70**

**-**	-	2 (0.3)	2 (0.3)	2 (0.3)	1 (0.2)	6 (0.5)
**ETO**	-	46 (1.1)	16 (0.8)	29 (1)	45 (1.1)	23 (0.9)
**ETO**	+	27 (1)^a^	17 (0.8)	17 (0.8)^a^	14 (0.77)^a^	7 (0.6)^a^
**CDDP**	-	48 (1.1)	21 (0.9)	32 (1)	62 (1)	78 (0.9)
**CDDP**	+	27 (1)^a^	9 (0.6)^a^	16 (0.8)^a^	25 (1)^a^	51 (1.1)^a^
**DOX**	-	29 (1)	15 (0.8)	14 (0.8)	26 (1)	49 (1.1)
**DOX**	+	16 (0.8)^a^	8 (0.6)^a^	6 (0.5)^a^	7 (0.6)^a^	13 (0.7)^a^
**VIN**	-	50 (1.1)	32 (1)	28 (1)	55 (1.1)	33 (1)
**VIN**	+	40 (1.1)^a^	17 (0.8)^a^	7 (0.6)^a^	46 (1.1)^a^	20 (0.9)^a^
**TAX**	-	37 (1.1)	16 (0.8)	20 (0.9)	32 (1)	27 (1)
**TAX**	+	16 (0.8)^a^	21 (0.9)	8 (0.6)^a^	17 (0.8)^a^	15 (0.8)^a^

^a^: P < 0.05 (student's paired t-test) as compared to cells cultured with drug alone

**Table 2:** Effect of ceramides on lymphoma and breast carcinoma cell lines apoptosis

**Culture conditions**		**% of apoptotic cell: Mean (SE)**						
		**LNH **					**breast carcinoma**	
**Drug**	**L cells CD40L**	**Daudi**	**Raji**	**BJAB**	**BL36**	**BL70**	**T47 D**	**BT20**

**-**	-	2 (0.3)	2 (0.3)	2 (0.3)	1 (0.2)	6 (0.5)	0 (0)	6 (0.5)
**C2**	-	24 (0.9)	64 (1)	13 (0.7)	64 (1)	67 (1)	29 (1)	13 (0.7)
**C2**	+	10 (0.7)^a^	17 (0.8)^a^	4 (0.4)^a^	42 (1)^a^	17 (0.8)^a^	8 (0.6)^a^	5 (0.5)^a^
**C6**	-	80 (0.9)	82 (0.9)	43 (1.1)	85 (0.8)	45 (1.1)	55 (1.1)	20 (0.9)
**C6**	+	59 (1.1)^a^	68 (1)^a^	12 (0.7)^a^	48 (1.1)^a^	20 (0.9)^a^	46 (1.1)	3 (0.4)^a^
**C(-)**	-	3 (0.4)	7 (0.57)	1 (0.2)	1 (0.2)	4 (0.4)	2 (0.3)	3 (0.3)

^a^: P < 0.05 (student's paired t-test) as compared with culture with ceramide alone

**Table 3:** Effect of ceramides on lymphoma and breast carcinoma cell lines proliferation

		**Culture conditions **Mean ^3^HTdR incorporation (SE)			
	**cells**	**C2 (50 μM)**	**C2 (50 μM) + CD40L L cells**	**C6 (50 μM)**	**C6 (50 μM) + CD40L L cells**

**T47D**	25451 (2967)	1573 (111)^a^	4768 (748)^b^	2113 (313)^a^	4782 (108)^b^
**BT20**	11005 (777)	916 (128)^a^	1144 (111)	1979 (184)^a^	3061 (284)^b^
**DAUDI**	38590 (1157)	15687 (473)^a^	34273 (1847)^b^	360 (65)^a^	2218 (906)
**RAJI**	120043 (3940)	59620 (4987)^a^	73050 (2168)^b^	1936 (965)^a^	6543 (1072)^b^
**BJAB**	147778 (25361)	101803 (1821)^a^	128084 (4793)^b^	18034 (920)^a^	47822 (2483)^b^
**BL36**	40653 (683)	10787 (3720)^a^	25756 (1322)^b^	215 (71)^a^	6544 (3106)^b^
**BL70**	131317 (7241)	42045 (2592)^a^	81700 (5781)^b^	568 (257)^a^	2636 (1103)

^a^: P < 0.05 (student's paired t-test) as compared to cells cultured without ceramide

^b^: P < 0.05 (student's paired t-test) as compared to cells cultured with ceramide alone

**Table 4:** Modulation of caspase-3/7 activity by drugs and CD40L

**Culture conditions**		**Caspase-3/7 Activity (pmol/min)**						
**Drug**	**L cells CD40L**	**Daudi**	**Raji**	**BJAB**	**BL36**	**BL70**	**T47 D**	**BT20**

**-**	-	14	10	3.2	15.5	10.3	1.4	2.1
**ETO**	-	45.7	40.8	45.9	35.4	25	2	5.5
**ETO**	+	17.5 ^a^	15.5^a^	13.4^a^	19.4^a^	10^a^	5.3	10.2
**CDDP**	-	21	34.6	88.6	19.1	19.5	0.9	2.2
**CDDP**	+	23	31.2	13.9^a^	32.6	24.5	4	1.5
**DOX**	-	34.8	36	10.8	37.9	23.5	3.2	8.5
**DOX**	+	12.3^a^	8.2^a^	1.2^a^	18.8^a^	5.1^a^	5.2	11.3
**VIN**	-	16.7	31.7	42.6	11.9	19	1.7	4.3
**VIN**	+	13.8	12.7^a^	6.5^a^	14.8	7.2^a^	4.8	10.1
**TAX**	-	29.7	30.6	39.2	21.2	20.4	2.3	5.4
**TAX**	+	13.1^a^	9.5^a^	8.5^a^	13^a^	4.5^a^	5.7	9.8

^a^: P < 0.05 (student's paired t-test) as compared to cells cultured with drug alone
